# Keel Bone Damage in Commercial Laying Hen Hybrids

**DOI:** 10.1002/vms3.70518

**Published:** 2025-07-24

**Authors:** N. Abdallah, K. Kursun, M. Baylan

**Affiliations:** ^1^ Department of Animal Science Faculty of Agriculture Çukurova University Adana Türkiye

**Keywords:** deviation, fracture, hens, housing, keel bone, welfare

## Abstract

This work reviews the influence of production systems, strain and age of laying hens on keel bone damages (KBDs) with the subsequent welfare implications and production losses. Damages to the keel bone are influenced to a greater extent by the type of production system and its complexity. Hens housed in cage‐free production systems are more likely to have a higher prevalence of this pathological condition than hens housed in cages. The aviaries, especially multi‐tier, are more associated with this pathological condition. Clumsy ascents or descents from perches and collisions during flight with objects in the poultry house are other factors influencing the risk of KBD. However, the strain and age of hens may also cause a variation in damages incurred by the keel bone with older hens more prone to KBD(s) than younger hens. Damages to the keel bone have a significant effect on the growth, production, behavioural and welfare performance of laying hens. The mobility of hens with KBD is more likely to be impaired leading to lower feed intake, egg production and poor eggshell quality. Hens with KBD may experience failure to reach nests, which may increase the number of floor eggs and microbial contamination of eggs. The effective use of perches and pop‐holes (in free range) may be limited among hens with severe damage to the keel bone. Hens with KBD may experience severe pain and discomfort leading to several hormonal and haematological changes. Impaired mobility may increase the time spent lying on litter substrates and thereby risk further damage to their feather and increase the risk of bumble foot/footpad dermatitis and breast blisters.

## Graphical abstract

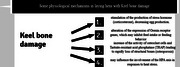

## 1 Introduction

One of the major welfare issues in layer chicken production is KBD, which also influences public opinion about commercial large‐scale egg production. In commercial laying hens, KBD is regarded as one of the important welfare problems (FAWC 2013; Tainika and Şekeroğlu 2020). Damages to the keel bone are described as a fracture (KF) or deviation depending on the type of damage. Riber et al. (2018) described KF as either sharp bends, shearing and/or fragmented sections of the keel bone. Additionally, Casey‐Trott et al. (2015) identified that KF can range from the cranial to the caudal, a combination of the two or from the ventral to the dorsal part of the keel. Additionally, KF is an indicator of poor welfare, and the high occurrence rates observed across different hen strains in many countries highlight the global significance of this topic (Riber et al. 2018). For instance, in Belgium, the Netherlands and the United Kingdom, the incidence of KF at the end of egg‐laying period between 86% and 97% among commercial flocks has been identified (Rodenburg et al. 2008; Wilkins et al. 2011).

Short‐term, highly energetic impacts during collisions with perches or other structures in the poultry unit may cause KF (Gregory et al. 1990). However, the development of a keel deformity is likely to occur over a period of time due to the bone remodelling itself in response to regular high pressure exerted on it (Scholz et al. 2008; Pickel et al. 2011). Käppeli, Gebhardt‐Henrich, Fröhlich, Pfulg, Schäublin, and Stoffel (2011) also observed a gradual increase in keel bone deformities with a prevalence rate of 35% and 43.8% in their first and second experiments, respectively.

The inclusion of perches in cages and non‐cage production systems has been identified as a major cause of KBD (Sandilands et al. 2009; Tainika and Şekeroğlu 2021). Furthermore, the complexity of the production system might increase the incidence and severity of damage to the keel bone. For example, collisions and sudden falls from perches and other platforms have been linked to the use of multi‐tier complex aviaries (Stratmann, Fröhlich, Gebhardt‐Henrich, et al. 2015), and these are likely to increase the incidence of damage to the keel bone (Makagon et al. 2017). However, Habig and Distl (2013) and Käppeli, Gebhardt‐Henrich, Fröhlich, Pfulg, and Stoffe (2011) determined that the age and strain of the hen also play a significant role in the prevalence and severity of damages to the keel bone.

Modern‐day laying hen strains have been selected for early reproductive maturity (age) and higher egg production (Tainika et al. 2024). However, during this time of sexual maturity, bone development is still ongoing. During the onset of laying, the majority of the calcium is allocated for the development of the eggshell rather than the continuous development of the bones. This leads to bone weakness known as osteoporosis, making them susceptible and fragile to breakages (Webster 2004; FAWC 2010). Understanding the association between estrogen, calcium and bone metabolism stages is very important in understanding KBD (Li et al. 2024). The functions of osteoclast and osteoblast are stimulated and inhibited by estrogen (Whitehead 2004; Bloom et al. 1958), respectively. However, the higher requirement for calcium at the peak of lay increased the activity of osteoclast and bone reabsorption. In addition, Z. Zhang et al. (2023) identified that this reabsorption is not confined to medullary bone, which explains the susceptibility of laying hens to keel damages. The dynamic balance between bone formation and reabsorption is significant in maintaining the quality of bones, and alterations in this balance due to the deficiency of calcium can lead to osteoporosis with increased fragility of the keel bone (Matsuo and Irie 2008).

KBD has been reported to decrease the secretion of orexin genes that stimulate feed intake, increase the production of stress‐related hormones (corticosterone) and reduce walking and standing behaviour in the affected hens (Wei et al. 2019; Wei, Feng, et al. 2022; Wei, Bi, et al. 2021; Casey‐Trott and Widowski 2016). All these factors lead to lower feed intake and blood levels of calcium, leading to a reduction in egg production, egg sizes and poorer eggshells. Additionally, a reduction in walking and standing behaviour and higher resting behaviour among hens with KBD may increase the duration of contact of the keel, hock and foot with soiled litter materials, increasing the risk of infection and the prevalence of breast blisters, hock burns and footpad dermatitis.

Therefore, this review aims to evaluate the impact of production systems, hen age and strain on KBD and their associated welfare implications.

### 1.1 Criteria for the Selection of Articles Used in This Review

The review is based on relevant scientific literature from the database ‘Google Scholar’ with ‘keel bone, fear, stress, behavior, egg production and egg quality’ as the main keywords (string) used. Priorities were given to research articles that have investigated KBD and its effect on production, behavior, fear and stress in laying hens. Additionally, only articles that have been peer‐reviewed and written in the English language were prioritised.

### 1.2 The Influence of Production Systems on KBD

The production systems for laying hens in the European Union and other developed regions of the world have been intensively examined since both cages and non‐cage production systems have been associated with the prevalence of KBD, which is one of the major welfare problems in laying hen production. For instance, the results of authors (Regmi et al. 2016; Fleming et al. 2004; Rodenburg et al. 2008; Wilkins et al. 2011; Riber and Hinrichsen 2016; Petrik et al. 2015) who have examined the incidence of KBD under different production systems revealed that the occurrence of this pathological condition may vary between 5% and 97% depending on the production system and the age of the laying hen. However, the prevalence of KBD among hens housed in non‐cage production systems has been identified to be higher, compared to hens housed in cages. For instance, the prevalence of KF among laying hens varies between 53% and 100% in the cage‐free systems, whereas its prevalence in enriched cages ranged between 50% and 98% (Thøfner et al. 2020). Additionally, in an on‐farm study, Petrik et al. (2015) identified a higher prevalence of KF among hens housed in the floor system (48.3% ± 0.04%), compared to hens housed in conventional cages (24.8% ± 0.03%) using the palpation method. Käppeli, Gebhardt‐Henrich, Fröhlich, Pfulg, and Stoffe (2011) also assessed KBD on a commercial farm using palpation and revealed that hens in commercial aviary houses (56.5%) were associated with a higher prevalence of total keel bone deformations, compared to those in floor/litter production system (44.0%). Moreover, a 67% prevalence of keel bone deviation with 3% fractures was reported by Göransson et al. (2023) in an organic laying hen farm. Additionally, the anatomical (dissection) examination of keel bones of hens housed in experimental units (small pens) by Freire et al. (2003) revealed the prevalence of KF between 70% and 97% among hens housed in litter/floor production system.

A palpation examination of the keel bone in commercial farms by Dedousi et al. (2020) revealed that the incidence of KBD was significantly different among hens housed in free‐range system (50%), enriched cages (24%) and floor system (7%), with 22% of the hens in the free‐range system having keel bone deformation, 7% having fractures and 21% having both deformation and fractures. However, a post‐mortem (dissection) examination of KBD on commercial farms by Sherwin et al. (2010) revealed that the incidence of recent or new KF was nearly five times more common in hens housed in traditional cages than hens in other production systems (furnished cages, free‐range and barn systems) used in their study. Habig et al. (2021) also examined the keel bone of hens housed in experimental units (small pens) using the palpation method and reported a higher prevalence of keel deformities but fewer KF in hens housed in cages than in hens housed in a litter system.

Furthermore, hens housed in the aviary system, especially the multi‐tier aviaries, are associated more with KBD than hens housed in single‐tier aviaries. For instance, a post‐mortem examination of the keel bone by Gretarsson et al. (2023) in commercial farms revealed that the prevalence of KF among hens housed in multitiered aviaries was 92%. Moreover, the prevalence of KBD was reported to be 73% in a multi‐tier aviary system (Freire et al. 2003), and a post‐mortem examination by Nicol et al. (2006) revealed that the incidence of KBD was 60% in hens housed in a commercial single‐tier aviary farm. Using the palpation method, Riber and Hinrichsen (2016) also observed a higher prevalence of KF among commercial flocks (hens) housed in multi‐tiered aviary system, compared to those housed in single‐tiered aviary system (11.6% vs. 4.9%, respectively). Additionally, a palpation examination of the keel bone in commercial hens revealed a higher KBD (fractures+deviations) in hens housed in commercial multitier aviaries than in those housed in single‐tier aviaries (Marggraff et al. 2024).

The higher prevalence of KBD among hens in non‐cage production systems is associated with the provision of extra spaces in these production systems for increased activity, and the addition of housing structures such as perches and platforms in the production systems has been identified as the major factors that increase the risk of collisions or injuries leading to susceptibility of the keel bone to breakages (Fleming et al. 2006; Scholz et al. 2009; Rodenburg et al. 2008; Wilkins et al. 2011; Käppeli, Gebhardt‐Henrich, Fröhlich, Pfulg, and Stoffe 2011). Furthermore, multi‐tier complex aviaries are associated with regular and severe falls and collisions, which are more likely to cause detrimental damage to the keel bone. Indeed, Baker et al. (2024) observed that the number of collisions with structures in the production system and the number of aggressive interactions affected the likelihood a hen may develop one or more KF within a 3‐week time period. Petrovič and Mellen (2024) also confirmed higher keel phosphorus, magnesium and calcium content in hens housed in enriched cages followed by those in the litter system with the least mineral contents (phosphorus, magnesium and calcium) identified in hens housed in the aviary system.

Contrary to the above findings, Casey‐Trott et al. (2017) examined the keel bone of hens in experimental pens using palpation and reported that hens housed in the aviary production system (41.6% ± 2.8%) had a lower prevalence of KF through the end‐of‐lay than those housed in conventional cages (60.3% ± 2.9%). The authors explained that the lower incidence of KF among hens in the aviary system is associated with the extra space in this production system, which encourages several exercises and movement that improve bone development and strength. Indeed, higher keel bone mineral density was observed in hens housed in aviaries than in hens housed in the litter system (Makagon et al. 2024).

The complexity of the production system such as the availability of enrichment structures including perches, platforms and other objects in cages and non‐cage production systems has been identified as one of the main causes of keel damage in laying hens. Some authors reported that hens may break their anatomically exposed keels when colliding with perches or other objects in alternative production systems (Tauson and Abrahamsson 1996; Fleming et al. 2004; Scholz et al. 2008; Abrahamsson et al. 1996; Gregory et al. 1990; Knowles and Wilkins 1998).

Wilkins et al. (2011) elaborated that uncontrolled ascents and descents from perches are likely to increase the prevalence of keel bone fracture. For instance, a lower prevalence of KF was reported at the end of lay for hens in conventional cages (83%), compared to those in cages with galvanised steel perches (92%; Hester et al. 2013). A 10%–34% increase in KF when perches were added to an organic production system was identified (Wilkins et al. 2011). The authors further reported that the inclusion of aerial perches increased the prevalence of KBD to 86%.

Whereas the presence of perch influences the prevalence of KBD, properties of the perch such as perch material, height and shape can also play a major role in the prevalence of this pathological condition (Wilkins et al. 2011; Pickel et al. 2011; Sandilands et al. 2009). For instance, Stratmann, Fröhlich, Harlander‐Matauschek, et al. (2015) examined the keel bones of hens using palpation and concluded that the provision of soft perches in experimental pens decreased the incidence of KF and deformities. It was also identified that the use of round metallic perches does not provide adequate support for grip (Pickel et al. 2010, 2011), and this could increase the number of unsuccessful landings on perches or sudden fall from perches. Perches made from wood and rubber materials have been reported to be better at absorbing forces during impacts and could prevent sudden falls from perches as well as reducing the prevalence of unsuccessful landings on perches. Cushioned/soft perches can minimise pressure load on the keel bone and increase its contact area when a hen is perching (Pickel et al. 2011), reducing the likelihood of damages to the keel. Additionally, Stratmann, Fröhlich, Harlander‐Matauschek, et al. (2015) speculated that it could be possible that during collisions, soft perches convert kinetic energy into deformation energy as the cushion is compressible and thus reduces the amount of energy the keel bone is required to absorb. The lack of shock absorption in the case of hard perches may cause a high level of energy to be transferred to the bone directly, which is likely to cause fractures (Alghamdi 2001). Assuming that a maximum contact area and a minimum peak force are essential for keel bones, this mechanism could explain the reduction in keel damages among hens housed with soft perch pens (Stratmann, Fröhlich, Harlander‐Matauschek, et al. 2015). Additionally, better body balance and grip could be achieved by increasing perch diameter (Pickel et al. 2010) resulting in less falls and collisions. Moreover, since much pressure is exerted on the keel bone during the initiation of flight behavior, the higher energy requirement to reach perches of higher height would lead to more pressure exerted on the keel, increasing the susceptibility of the keel bone to damages.

Perch shapes could also have the potential to reduce localised pressure on the foot pad and the keel, reducing the incidence of bumble foot and KBD. Indeed, using the palpation method, DePaoli et al. (2024) identified more severe KBD among hens housed in experimental units with round perches in comparison to those housed with mushroom perches. The authors concluded that mushroom perches are wider than round perches and more evenly distribute the force exerted on the keel, leading to a lower peak force exerted on the keel. The summary of findings reporting the influence of housing system and its complexity on the prevalence (%) of KBD is given in Table 1.

**TABLE 1:** Summary of findings reporting the influence of housing system and its complexity on keel bone damage (KBD; %).

Authors		Housing systems and designs		Mean ±SEM	*p‐*values	Method of assessment	Study setting
Petrik et al. (2015)		Floor		48.3 ± 0.04	< 0.001	Palpation	Commercial farm
		Cage		24.8 ± 0.03			
Käppeli, Gebhardt‐Henrich, Fröhlich, Pfulg, and Stoffe (2011)		Floor		44 ± 0.07	< 0.05	Palpation	Commercial farm
		Aviary		56.5 ± 0.03			
Thøfner et al. (2020)		Cage		4.00	0.001	Computed tomography scan	Commercial farm
		Litter		27.00			
Sherwin et al. (2010)	Hens with recent KF	Conventional cages		24.6 ± 3.8	< 0.001	Dissection	Commercial farm
		Furnished Cages		3.6 ± 2.7			
		Ban		1.2 ± 2.7			
		Free‐range		1.3 ± 3.8			
	Hens with old KF	Conventional cages		17.7 ± 6.1	< 0.001		
		Furnished Cages		31.7 ± 4.3			
		Ban		69.1 ± 4.3			
		Free‐range		59.8 ± 6.1			
Dedousi et al. (2020)		Free‐range		50.00	Unspecified	Palpation	Commercial farms
		Enriched Cages		24.00			
		Litter		7.00			
Stratmann, Fröhlich, Harlander‐Matauschek, et al. (2015)		Aviary—with soft perches		15.4	0.0012	Palpation	Commercial laying hen houses
		Aviary—with metallic perches		21.5			
Casey‐Trott et al. (2017)		Conventional cages		60.3 ± 2.9	< 0.001	Palpation	Research farm
		Aviary		41.5 ± 2.8			
Heerkens, Delezie, Rodenburg, et al. (2016)		Portal‐type aviaries		55.6	0.005	Palpation	Commercial farm
		Row‐type aviaries		68.3			
		Aviaries—with plastic slatted floor		76.1	0.003		
		Aviaries–with wire mesh floor		85.5			
Hester et al. (2013)		KF	Cage—without perches	88	0.96	Dissection	Research farm
			Cage‐with perches	88			
		KS	Cage—without perches	3.15	0.49		
			Cage‐with perches	3.08			
Käppeli, Gebhardt‐Henrich, Fröhlich, Pfulg, Schäublin, and Stoffel (2011)			Litter system—with plastic perches	Metallic perches ↑ KF	<0.0001	Palpation	Research farm
			Litter system—with metallic perches				
Makagon et al. (2024)			Litter system—with perches	0.96	Unspecified	Radiography	Research farm
			Single‐tiered aviary	0.95			
			2‐tiered aviary	0.94			
Tauson and Abrahamsson (1996)			Conventional cages	3.97	< 0.05	Unidentified	Research farm
			Cages—with rubber perches	3.81			
			Cages—with hardwood perches	3.85			
Saraiva et al. (2019)		Compression	Ban	32.4	< 0.000	Visual inspection	Commercial farm
			Free‐range	28.7			
			Furnished cage	48.3			
		Moderate deviation	Ban	28.5	< 0.000		
			Free‐range	47.3			
			Furnished cage	24.9			
		Severe deviation	Ban	39.2	< 0.000		
			Free‐range	24.1			
			Furnished cage	26.8			

Abbreviations: KF (keel fracture), KS (keel score), ↑ (increase).

Although hens housed in cages have reduced bone strength (Fleming et al. 2006; Knowles and Wilkins 1998; Newman and Leeson 1998), the prevalence of KBD is higher when production systems are equipped with perches irrespective of the superior bone‐breaking strength of hens kept in alternative production systems. Thus, collisions and landing accidents are likely to play a significant role in the aetiology of keel bone trauma (Appleby et al. 1993; Tauson and Abrahamsson 1994). Although the use of perches is known to improve bone mineralisation, it was reported that increased bone mineralisation of the keel due to access to perches at rearing and egg‐laying phases did not prevent the higher prevalence of keel fractures at the end of the egg‐laying phase (Hester et al. 2013).

In summary, a higher prevalence of KBD in hens housed in non‐cage production systems, compared to hens housed in cages, is more likely to be identified. The use of enrichment structures such as perches and platforms and the provision of extra spaces for increased activity in the non‐cage production systems and enriched cages is associated with better welfare. However, uncontrolled ascent and descent from perches and platforms and collisions among hens or with housing structures are likely to increase the prevalence and severity of damages to the keel bone. Additionally, among the non‐cage production systems, hens housed in aviaries, especially the multi‐tier aviaries, are at higher risk of developing KBDs.

### 1.3 The Influence of Hen Age and Strain on Damages to the Keel Bone

It has been known to researchers that the age and the strain of the hen may influence the severity and the incidence of damages to the keel. For instance, Eusemann et al. (2020) reported that KF is closely linked to the age of the hen. Different studies (Rufener, Abreu, et al. 2019; Rufener, Baur, et al. 2019; Wei et al. 2020) have also confirmed that the age of the laying hen influenced the prevalence of damage to the keel bone. Harlander‐Matauschek et al. (2015) identified a higher prevalence of KBD between the onset and peak of lay. Toscano et al. (2018) also identified that the stability of the keel bone was achieved after 49 weeks of age in an experimentally induced postmortem (dissection) fractures. Similarly, Rufener and Makong (2020) also observed an increased prevalence of KF from the onset to the end of lay; however, after 49 weeks of age, the prevalence of KF levelled. A palpation inspection of the keel revealed a higher prevalence of KBD among hens at 62 weeks of age, compared to 32 weeks (Riber and Hinrichsen 2016). Furthermore, the palpation results of Marggraff et al. (2024) revealed that the prevalence of KF and deviation was between 26% and 74% at 65–74 weeks of age. Using quantitative computed tomography (CT) scans, Emmert et al. (2024) identified that 80% of the hens in their study at 16 weeks of age had no signs of KBD, while 20% of the hen had mild KBD. Moreover, at 52 weeks of age, 31% of the hens had mild KBD, 61% with moderate KBD and 8% with severe damages to their keel bone. The radiography examination of the keel bone by Makagon et al. (2024) revealed changes in KF with a gradual rise between 26 and 28 weeks of age and the most severe fracture determined at 30 weeks of age.

The higher prevalence or severity of KBD observed in older hens could be attributed to bone weakness due to old age. As hens age, there are difficulties in nutrient utilisation, which lead to progressive bone weakness. Bones that are already weak or have lost their strength are more prone to further damage. Additionally, during the onset of lay (around 18–20 weeks of age), the keel bone is not fully developed; however, the majority of the calcium is allocated to eggshell formation, leaving the keel prone to damage. Johnson (2015) stated that to cover the high demand of calcium for eggshell formation, laying hens mobilise 40%–60% of their daily calcium requirement from bones. As egg production continues, there is a gradual reduction in the structural bone resulting in a progressive weakening of bones and thus increasing bone fragility to fracture (Whitehead 2004). Because commercial laying hen strains have been selected for early reproductive maturity (age) and higher egg production, the severity of keel bone may vary with the age at which the hen started laying eggs. Additionally, it was reported that the ossification of the keel is not fully completed until 30–40 weeks of age (Eusemann et al. 2020) indicating that hens with earlier reproductive age (15–20 weeks of age) before the keel bone is fully mature are more likely to have a high prevalence of KF with the caudal part being the most affected area by fractures (Thøfner et al. 2020; Baur et al. 2020).

Commercial laying hen hybrids differ in terms of production performance and bone‐breaking strength. For instance, lower egg production has been reported in brown‐laying hens, compared to white‐laying hens (Rufener and Makong 2020) and higher egg production is positively correlated with higher calcium requirement for eggshell formation. Indeed, a palpation examination of the keel bone by Marggraff et al. (2024) also revealed a higher KBD in white‐laying hen strains than brown strains. Therefore, it is likely that the prevalence of KBD may be higher in highly productive strains. Habig et al. (2021), using the palpation method, reported a higher prevalence of keel bone deformity and lower keel mineral density in white layers than in brown layers. Wei, Feng, et al. (2022) examined the incidence of KF and deviation using an X‐ray and a palpation method, respectively. The authors identified a higher prevalence of KF in Lindian chickens (34%), compared to Hy‐Line Brown (21%); however, the prevalence of keel deformities was higher in Hy‐Line Brown (27%) than in Lindian chickens (22%). Kittelsen et al. (2021) also examined the keel bone of hens using the palpation and the inspection methods. The authors reported that the percentage of hens without KF was lower among white leghorn hens (31%), compared to red jungle fowl hens (90%).

Although the correlation between KBD and high egg production has been demonstrated, other studies examining the prevalence of KF across strains have reported higher KF and deviations in brown strains than white strains (Vits et al. 2005; Habig and Distl 2013; Heerkens, Delezie, Ampe, et al. 2016; Candelotto et al. 2017; Gebhardt‐Henrich et al. 2017; Eusemann et al. 2018; DePaoli et al. 2024). Additionally, higher prevalence of KBD (fracture, thickening and deviation) in Hy‐Line Brown than Isa Tinted hens was identified (Uysal et al. 2024). Gebhardt‐Henrich et al. (2017) explained that the higher incidence of KBD among brown hens than white hens could perhaps be due to the higher body weights of the brown hen strain, compared to white layers. During the initiation of flight behaviour in hens, the body weight is supported by the keel bone, so birds with heavier body weight will have more pressure exerted on their keel bone. The variations in bone‐breaking strength as a result of genotypic differences may also play a role in the aetiology of the keel bone.

In conclusion, the prevalence of KBD may be influenced by both the age and the strain of the hen, and it is more likely that highly productive strains may be more prone to bone weakness due to the profound requirement of calcium for egg production. Additionally, strains with heavier body weight may be more susceptible to KBD due to high pressure on the keel bone as a result of regular flight behaviour. Furthermore, hens with early sexual maturity age at the initial stage of the keel bone development might be more prone to keel injuries.

### 1.4 Relationship Between KBD and the Behavior of the Affected Hen

Damages to the keel bone have been reported to influence the behavior of the affected hens. Riber et al. (2018) reported that the keel bone is the site of the muscle attachment for flight, and therefore any damages to the keel bone may impair several locomotive behaviors. It was reported by some authors (Nasr et al. 2012; Casey‐Trott and Widowski 2016) that hens with KF spent more time sleeping on the floor and failed to reach perches of 100 cm height. Gebhardt‐Henrich and Frohlich (2015) also observed that the time spent in the nest after egg‐laying was higher among hens with KF, and the authors speculated that difficulty in egg laying could have caused the hens to spend more time resting in nests. Riber et al. (2018) identified lower standing behavior and higher perching time in hens with KF as compared to their counterparts with little to no fractures. The presence of visible KF has also been reported to decrease perching behavior, nest and pop‐hole accessibility (Rentsch et al. 2019; Richards et al. 2012). Furthermore, Richards et al. (2012) elaborated that the use of pop‐holes or outdoor environments at 45 weeks of age was 54% by hens with non‐fractured keels; however, the usage was 35% and 11% at 45 weeks of age among hens with minor and severely fractured keels, respectively. KBD has been reported to cause severe distress and pain, and therefore hens with severe keel damage may reduce any energetic activity to avoid unnecessary discomfort associated with movements. Other authors (Wei et al. 2020; Wei, Feng, et al. 2022) reported that hens with severe keel bone fracture had a lower duration to react to a novel object as well as a high duration of tonic immobility duration indicating that KBD is likely to increase fear in affected hens. Summary of some behavioral changes due to keel damage is presented in Table 2.

**TABLE 2:** Summary of findings reporting the influence of KBD on behavioral responses of affected hens.

Outcomes	*p*‐values	Authors
↓ Access perch height 50 cm	NS	Nasr et al. (2012)
↓ Flying from ground to perch 100 cm height	NS	
↑ Landing from 50 cm perch height to floor	< 0.05	
↓ Landing from 100 cm perch height to floor	< 0.01	
↑ Average of time taken to reach food	< 0.05	R. Zhang et al. (2021)
↑ Landing from 150 cm height perch to floor	NS	
↑ Latency of novel object test	< 0.01	
↑ Duration of tonic immobility	< 0.01	
↓ Number of wing flapping during inversion test	0.01	
↓ Wing flapping duration during inversion test	0.12	
↑ Standing	0.001	Wei et al. (2020)
↓ Perching	0.015	
↓ Walking	0.001	
↓ Jumping	0.004	
↑ Sitting	0.025	
↑ Duration of tonic immobility	0.025	
↓ Latency to approach a novel object	0.002	Wei, Feng, et al. (2022)
↑ Human approach test	0.108	
↑ Duration of tonic immobility	< 0.001	
↓ Tonic immobility induction number	0.782	
↑ Rest on perch	0.0114	Casey‐Trott and Widowski (2016)
↓ Rest on floor	0.0161	
↓ Standing	< 0.0001	
↑ Preening	0.2116	
↑ Sitting	0.4744	
↓ Dustbathing	0.9559	
Walking pace on ramps	0.29	Rentsch et al. (2019)
↓ Pecking	0.052	Rokavec and Zupan Šemrov (2020)
↑ Latency to leave the start box	0.01	
↑ Crossing central zone	0.02	
↓ Standing	≤ 0.05	Wei, Bi, et al. (2021)
↓ Walking	≤ 0.05	
↓ Perching	≤ 0.05	
↑ Sitting	≤ 0.05	
↓ Jumping	≤ 0.05	
↑ Tonic immobility duration	≤ 0.05	

*Note*: ↓ (decrease or lower); ↑ (increase or higher), NS (not significant).

In summary, KBD may cause several disturbances that limit the normal and daily behavioral activities of the affected animal since movement may lead to severe pain and distress.

### 1.5 The Effect of KBD on Physiological Changes in the Affected Hens

The influence of KBD may not just be limited to physical hindrance but also to physiological impairments. For instance, higher temperature around the keel region of hens with intact keels than hens with fractured keels was reported by Nasr et al. (2012), and the authors speculated that this could be due to the disuse and atrophy of the breast muscle filets or tenders. KBD may have detrimental effects on the metabolism and thermoregulation because the keel is also reported to be the site of attachment for the muscles involved in inhalation and exhalation (Codd et al. 2005). Due to the lack of a muscular diaphragm in avian species, they depend on the oscillation of the keel bone and ribs to drive inhalation and exhalation (Claessens 2009). This means that any damage to the keel bone could impair the involvement of the bone in respiration and could hinder the thermoregulatory mechanism of hens (Riber et al. 2018).

The postmortem examinations of the keel bone revealed variations in the breaking strength and mineral density of hens with fractured or deformed keels, compared to their counterparts with non‐fractured/deformed keels. For instance, Gebhardt‐Henrich et al. (2017) reported a higher keel bone shearing strength of hens with normal keels than their counterparts with fractured keels. Higher keel calcium composition in hens with normal or slightly deviated keels, compared to hens with fractured keels, was also determined (Gebhardt‐Henrich et al. 2017). Similarly, other authors (Nasr et al. 2012, 2013 **; **Toscano et al. 2013) also reported a reduction in keel mineral density and keel bone strength of laying hens with KF, compared to hens without KF. Furthermore, lower Na, P and Ca contents coupled with higher levels of osteocalcin and increased activity of alkaline phosphatase and tartrate‐resistant acid phosphatase (TRAP) have been reported in hens with keel damages (Wei, Bi, et al. 2021). High levels of serum TRAP activity (Janckila and Yam 2009) and osteocalcin increase the rate of bone deterioration or disorder, indicating that the presence of any damages to the keel bone could further increase the rate of deterioration of the keel due to increased TRAP and osteocalcin activities. The findings of Wei, Feng, et al. (2022) revealed that hens with fractured keels had higher blood corticosterone levels, serum interleukin (IL)‐1β and IL‐6 but lower levels of serotonin than hens with non‐fractured or deformed keels. The stimulation of glucocorticoids including corticosterone has been reported to participate in the regulation of neurogenesis, dendritic remodelling and long‐term depression in the hippocampus and other limbic structures (McEwen 2000; Sapolsky 2003), which can in turn alter brain function and increase vulnerability to neurological impacts. IL‐1β and IL‐6 are involved in the reaction of emotions, such as anxiety and fear in animals and humans, and positive emotion is related to decreased levels of proinflammatory cytokines (Anisman et al. 2008; Song and Wang 2011). It is therefore speculated that the secretion of IL‐1β and IL‐6 could cause negative emotions, leading to stress. Serotonin on the other hand influences the mechanisms that improve the positive affective state of animals, and higher blood levels of this hormone are associated with a reduction in stress and fear.

Studies have not entirely focused on the influence of KBD on genomic pathways; however, Wei et al. (2019) reported decreased orexin and orexin receptor genes, increased stress and inflammatory responses in hens with KBD. The orexin neurons are localised in an area (lateral hypothalamic) that has been identified to be involved in energy homeostasis and the control of food intake. For instance, in energy‐restricted dairy cows, Orexin‐A neurons are colocalised with adenosine monophosphate‐activated protein kinase (AMPK) and peroxisome proliferator‐activated receptor (PPAR) gamma (Ramser and Dridi 2022). Additionally, energy restriction phosphorylates AMPK leading to AMPK's activation and to an increased PPAR expression, indicating orexin‐A's control of energy homeostasis (Kuhla et al. 2011). AMPK is an enzyme that acts as a sensor for cellular energy status through changes in the ATP‐to‐AMP ratio and activating downstream targets to stimulate metabolic shifts (O'neill 2013; Herzig and Shaw 2018). Herzig and Shaw (2018) reported that fat metabolism, glucose utilisation and the balance between anabolic and catabolic pathways within cells are influenced by AMPK. Since birds eat to meet their energy requirement, any physical or physiological stimuli that may cause imbalances in the orexin and orexin gene receptors could alter the metabolic pathway affecting the mechanisms (e.g., glucostatic, thermostatic and aminostatic mechanisms and gastric distension) that influences negative feedback and voluntary feed intake in farm animals.

Additionally, transcriptome sequencing analysis of normal and fractured keels identified 214 differentially expressed genes (DEGs), among which 88 were upregulated and 126 downregulated (Wei et al. 2023). The direct effect of KBD on candidate genes essential for growth and egg production is not known yet; however, the stress associated with KBD may inhibit the activity or the mechanism involved in the activation and stimulation of candidate genes necessary for growth and reproduction.

Additionally, it was identified that the prevalence of keel damage decreased the strength and ash component of the tibia bone, and this suggests that the strength of other bones may influence the susceptibility of the keel bone to damages (Donaldson et al. 2012).

Furthermore, the results of Wei et al. (2023) revealed that the concentrations of osteocalcin, calcitonin, 25‐hydroxyvitamin D3, phosphorus and Ca, as well as the activities of TRAP and alkaline phosphatase in serum samples of hens with fractured keels, were lower than those with non‐fractured keels. The authors further identified that among hens with fractured keels, the parathyroid hormone, osteoprotegerin and corticosterone concentrations were higher than those of the hens with non‐fractured keels.

Calcium and phosphorus are the main structural components of bones, and decreased levels of these minerals may result in bone weakness. Furthermore, calcitonin and parathyroid hormone are the main hormones that regulate blood levels of calcium. Any stimuli that cause physiological imbalances of these hormones may further inhibit their activities leading to a reduction in bone strength. Table 3 provides a summary of the influence of KBD on some physiological changes in hens.

**TABLE 3:** The influence of KBD on physiological responses of the affected hens.

Outcomes	*p*‐values	Authors
↓ Mineral density of bone	< 0.001	Habig et al. (2021)
↓ Ash weight (g)	< 0.001	
↓ Bone breaking strength	< 0.05	Donaldson et al. (2012)
↑ Bone diameter	NS	
↓ Bone weight	NS	
↓ Blood serotonin level	0.009	Wei, Feng, et al. (2022)
↑ Blood corticosterone	< 0.001	
↑ Serum interleukin‐1β	< 0.001	
↑ Serum interleukin‐6	< 0.001	
↓ Keel strength	< 0.01	Nasr et al. (2012)
↓ Temperature around the area of the keel	< 0.01	
↓ Keel strength	< 0.000	Nasr et al. (2013)
↓ Keel strength	< 0.0001	Gebhardt‐Henrich et al. (2017)
↓ Keel calcium content	< 0.04	
↓ Orexin	< 0.05	Wei et al. (2019)
↑ Corticosterone	< 0.05	
↑ Inflammation	< 0.05	
↑ Hsp70 and Hsp90	< 0.05	
↓ Na	< 0.05	Wei, Chen, et al. (2021)
↓ P	< 0.05	
↓ Ca	< 0.05	
↑ Levels of osteocalcin	< 0.01	
↑ Alkaline phosphatase activity	< 0.05	
↑ Tartrate‐resistant acid phosphatase (TRAP) activity	< 0.01	
↑ Corticosterone	< 0.001	R. Zhang et al. (2021)
↓ Serotonin	0.017	
↑ Serum interleukin‐1β	< 0.001	
↑ Serum interleukin‐6	< 0.001	

*Note*: Na (sodium): Ca (calcium): P (phosphorus); ↓ (decrease or lower); ↑ (Increase or higher).

It was therefore concluded that any damages to the keel bone may cause severe pain and distress leading to several physiological imbalances. For instance, stress has been identified to increase the secretion of corticosterone, which in turn has a negative effect on hormone‐related production and reproductive performance as well as modification of behavioural activities. However, the degree of these physiological imbalances may depend on the severity of the damage to the keel bone.

### 1.6 The Effect of KBD on Production Parameters

The various behavioral and physiological changes caused by the damage to the keel bone may further have a huge negative effect on production parameters either directly or indirectly. The association between KBD and its effect on production parameters has been reported by several authors. For instance, in a study conducted by Wei et al. (2020), laying hens with KBD had lower body weight, feed intake, egg production and poor eggshell quality. The authors explained that the stress and impaired mobility as a result of damages to the keel may have caused the decreased feeding behaviour, which in turn affected production and egg quality traits. Furthermore, the reduction in the expression of orexin and orexin receptor genes due to stress and negative emotions associated with keel damages may inhibit feed intake or feeding behaviour (Greene et al. 2016; Nguyen et al. 2017). Reduction in egg production among hens with fractured keels, compared to hens with normal keels, has been observed. However, egg size was larger in the former, compared to the latter (Rufener, Baur, et al. 2019). Furthermore, it has been identified that hens with fractured keels had lower egg production, smaller egg sizes coupled with smaller surface area and poor eggshell quality (Nasr et al. 2012; Candelotto et al. 2017; Abdallah et al. 2022).

Contrary to the feeding behaviour observed by Wei et al. (2020) in hens with fractured keels, Nasr et al. (2013) observed a higher feed and water intake among hens with fractured keels. The authors explained that it may be possible that hens with fractured keels needed more calcium for the healing process, or they were severely hungry due to a decreased ability to access the feed and water regularly because of the impairment of mobility. The higher water intake of hens with fractured keels may simply be due to their increased feed intake or could also be a reflection of stress (Nasr et al. 2013) since chickens under stress conditions have been reported to have higher water consumption. Additionally, the poor eggshell quality among laying hens with fractured keels could be related to their calcium intake. For instance, each eggshell contains up to 3 g of calcium (Roberts (2004), and therefore laying hens need to absorb 3 g of calcium per day from feed for optimum eggshell formation, but under severe KBD conditions, mobility is inhibited, which causes a reduction in feed intake and subsequent reduction in daily intake of calcium thereby affecting the availability of calcium for egg production and eggshell formation. This may result in poor eggshell strength and thickness. It is also assumed that hens with KBD may use the majority of the calcium from feed for healing the broken bone rather than egg production or eggshell formation.

According to Gebhardt‐Henrich and Frohlich (2015), early initiation of egg production was positively correlated with the presence of KF, but no correlations were observed between the total egg production and the presence of KF. Edgar et al. (2023) also observed that hens with non‐fractured keels were lighter and laid eggs with less eggshell membrane, compared to hens with healed fractured keel bones; however, no differences were found between the two groups in terms of other production traits.

Severe KBD could have negative implications on meat quality, decreasing farmers’ profits by reducing the carcass value, especially in countries where laying hens are used for human consumption. The summary of the results of some studies on the effect of KBD on production parameters is shown in Table 4.

**TABLE 4:** Simplified summary of previous research reporting main findings of the influence of keel bone damage on production parameters.

Outcomes	p‐values	Authors
↓ Eggshell strength	0.031	Wei et al. (2020)
↓ Egg production	0.023	
↓ Eggshell thickness	0.001	
↓ Eggshell weight	0.051	
↓ Egg surface area	0.040	
↓ Haugh unit	0.049	
↓ Body weight	0.024	
↓ Feed intake	0.031	
↓ Number of eggs	—	
↑ Broken egg rate	0.004	
↑ Dirty egg rate	0.103	
↑ Feed intake	0.04	Nasr et al. (2013)
↑ Water intake	0.03	
↓ Egg weight	0.03	
↓ Body weight	0.37	
Earlier first egg appearance	0.04	Gebhardt‐Henrich and Frohlich (2015)
↓ Eggshell strength	0.060	Candelotto et al. (2017)
↓ Shell width	0.038	
↓ Eggshell weight	NS	Nasr et al. (2012)
↓ Egg surface area	NS	
↓ Egg production	< 0.01	
↓ Shell percentage	NS	
↓ Shell density	NS	
↑ Shape index	NS	
↑ Egg production, ↓ daily feed intake, ↓ egg weight, ↓ eggshell thickness, ↑ broken‐egg rate	≤ 0.05	Wei, Bi, et al. (2021)

*Note*: ↓ (decrease or lower); ↑ (increase or higher).

It is therefore concluded that severe damage to the keel bone may have a huge negative impact on production parameters due to both physical and physiological limitations on feed intake, egg production and nutrient utilisation; however, the degree of this impact may vary depending on the severity of the damages to the keel bone.

### 1.7 Clinical Welfare Indicators Associated With KBD

KBD itself is a clinical condition associated with severe pains and impairments of both physiological and physical activities. For example, a postmortem examination of dead or culled hens from three non‐cage US farms by Kajlich et al. (2016) revealed that over 40% of the dead or culled hens had a deviated keel bone. In a study conducted by Donaldson et al. (2012), the authors reported that feather coverage tended to decline with increasing keel damage in the affected hens. Additionally, Marggraff et al. (2024) reported an increasing trend for feather damage with an increase in the severity of KBD. Although very little is known about how keel damage affects or interacts with other clinical conditions, the presence of KBD is an indicator of reduced welfare, and there seems to be relations between KBD and other clinical indicators of reduced welfare such as bumble foot (Riber et al. 2018).

It is therefore assumed that hens with severely damaged keels may spend more time lying on the ground increasing the time of contact of the breast, foot and hocks with the litter. This may increase the risk of exposure to agents causing footpad dermatitis, breast blisters and hock burns. Furthermore, hens with KBD are reported to be stressed, and stress is a major cause of poor feather coverage among hens. Another factor that may cause feather problems observed in hens with KBD could be the lack of good nutrition due to poor feed intake and nutrient utilisation. Additionally, hens with damaged keels may likely have increased body contact with the ground, and in poultry houses with poor litter conditions, this may affect plumage quality and also increases the incidence or likelihood of pathogenic infections. It is encouraged that future research should focus on the relationship between KBD and clinical welfare conditions to provide substantial evidence on this topic.

### 1.8 Management and Healing of KBD

KBD has been associated with the housing systems and their complexity. Reduction in failed landing attempts on perches and platforms and collision with enrichment structures in the production systems is a possible means of reducing the incidence of KBD among hens. For instance, providing ramps between perches has successfully reduced KF in hens (Heerkens, Delezie, Ampe, et al. 2016). Stratmann, Fröhlich, Harlander‐Matauschek, et al. (2015) also confirmed 44% more controlled movements and 45% and 59% fewer falls and collisions in aviary systems with ramps. Additionally, the authors reported 47% more controlled movements in aviary systems equipped with platforms and fewer KF in houses with ramps, compared to the control pens. Additionally, the provision of adequate stocking density (space) per bird could reduce stress and aid movement. This could also reduce stress‐associated agonistic behaviour (Abdallah et al. 2024) and physical altercations, reducing the likelihood of injuries suffered by the keel. The provision of enrichments such as dust baths, foraging substrates and pecking blocks could reduce boredom and stress, which are often precursors to aggressive interactions that can lead to physical harm (Baker et al. 2024).

Nutritional strategies such as the supplementation of calcium and phosphorus could serve as a possible means of managing and handling KBD. Indeed, the incidence of KBD was identified as higher in a hens‐fed diet containing 0.15% phosphorus than in a hens‐fed diet containing 0.3% P (Wei, Bi, et al. 2022). Habig et al. (2021) identified that hens with lower keel mineral density were associated with keel deformities. Additionally, the supplementation of omega‐3 fatty acids could serve as a potential means of solving keel‐related injuries in hens.

Genetic selection for specific bone traits has shown superiority in improving keel bone strength and reducing the incidence of keel damage. Indeed, Stratmann et al. (2016) concluded that the selection of specific bone traits associated with bone strength as well as the related differences in body morphology (i.e., lower index of wing loading) has the potential to reduce KBD in laying hens. Poor humerus and tibia breaking strength and the density of the whole keel bone have also been associated with the prevalence of KBD (Maidin et al. 2024), suggesting that hens with poor skeletal quality are likely to be prone to keel injuries. Additionally, the pullet rearing stage is a period of substantial musculoskeletal growth, and providing opportunities for exercise during this stage offers a proactive approach to reducing osteoporosis by stimulating osteogenesis (Casey‐Trott 2016). This approach could serve as a means of enhancing skeletal development in hens before the onset of egg production.

There is no substantial evidence on the mechanism that is involved in the healing process of the keel bone; however, in brief, the mechanism of bone (fracture) healing involves three different stages: inflammatory, repair and remodelling. The inflammatory phase begins immediately after fracture and occurs for approximately the first 2 to 3 weeks after injury (Eurell and Sickle 1998). Lysosomal enzyme released by dead osteocytes triggers the destruction of the organic matrix, and proteins such as IL‐1 and IL‐6 (Mountziaris and Mikos 2008) activate proteolytic enzyme cascades, resulting in coagulation and further inflammation. Platelets release platelet‐derived growth factor (PDGF), TGFI3 and epidermal growth factor, which are molecular mediators required for healing (Remedios 1999). Haematoma formation provides the first population of cells to the fracture site, including granulocytes, macrophages, lymphocytes and mast cells (Ashton et al. 1980). Osteoclasts, or derived mesenchymal cell lines, are found early in inflammation and begin the process of resorption and removal of dead bone (Remedios 1999). Endothelial cells also contain endothelial cell‐derived growth factors, which cause bone cell proliferation (Sorgente and Dorey 1980). Osteoblasts produce woven bone that is random in orientation and interwoven with the capillary channels. At this stage, the bone union is achieved, but the fracture site is structurally different from that of the original bone.

### 1.9 Assessment of KBD

Currently, KBD is assessed using palpation, anatomy, CT scanning and radiography. The methods vary significantly in terms of their accuracy, sensitivity and reliability, making it difficult to understand the extent and prevalence of these damages. However, palpation is the most widely used method due to its ease of adoption, low cost and the method being well‐validated (Li et al. 2024). However, other alternative methods should be adopted since they can offer further insight into KBD, which is not possible with palpation. Comparing the studies that have used palpation to assess the prevalence of KBD to other studies that have used different assessment methods (in Table 1), we realised that the average value for the prevalence of KBD was higher using palpation (prevalence rate of 45.25% for palpation and 28% for other assessment methods). This could be largely associated with the inaccuracy of this method (palpation) in identifying the actual prevalence rate of KBD.

Additionally, comparing the studies that have assessed the prevalence of KBD on commercial farms to those that have assessed the prevalence of KBD in research units/small pens (Table 1), we realised that the prevalence of KBD was higher in commercial farms (34.9%) than in small pens (24.9%). The higher prevalence of KBD in commercial farms could be associated with the complexity of the housing units on farms coupled with high stocking densities.

### 1.10 Future Research

During this literature review, we realised that research has not focused on the effect of KBD on genes and hormones associated with growth and reproduction, making this area an interesting area for further studies. Additionally, detailed insight into how keel injuries interact with the mechanism that activates the stimulation of reproductive hormones, oviduct and follicular development needs to be studied. Interestingly, the healing process of the keel bone during the rearing and laying stages has not been fully studied. Understanding the mechanism involved in the healing process of the keel could help facilitate the healing process.

## 2 Conclusion

In conclusion, KBD represents a welfare and productivity problem for the laying hen industry, and the prevalence of this condition may be more common in non‐cage housing systems, with highly productive hen strains and older hens being likely to be more susceptible. The use of soft perches and ramps could serve as a potential strategy in minimising falls and collisions, and hence reducing the incidence of KBD. The manipulation of dietary calcium and phosphorus levels during the rearing and egg‐laying phase and genetic selection for parents with superior keel and general bone strength could be very beneficial in tackling keel‐related problems in hens. Additionally, using welfare monitoring technologies to locate specific behaviours and impacts in pens that may likely lead to keel injuries could be very useful. Managing stocking density and using specific enrichments that promote specific behaviours could minimise aggressive interactions, reducing the likelihood of injuries sustained to the keel.

## Author Contributions

Conceptualisation: Abdallah N and M. Baylan. Writing—original draft: N. Abdallah. Writing—review and editing: N. Abdallah and K. Kursun.

## Ethics Statement

The authors have nothing to report.

## Conflicts of Interest

The authors declare no conflicts of interest.

## Peer Review

The peer review history for this article is available at https://publons.com/publon/10.1002/vms3.70518.

## Data Availability

The data for this study is available with the corresponding author and will be shared upon request.
